# Assessing the evolutionary rate of positional orthologous genes in prokaryotes using synteny data

**DOI:** 10.1186/1471-2148-7-237

**Published:** 2007-11-29

**Authors:** Frédéric Lemoine, Olivier Lespinet, Bernard Labedan

**Affiliations:** 1 Institut de Génétique et Microbiologie, CNRS UMR 8621, Bâtiment 400, Université Paris Sud XI, 91405 Orsay Cedex, France; 2 Laboratoire de Recherche en Informatique, CNRS UMR 8623, Bâtiment 490, Université Paris Sud XI, 91405 Orsay Cedex, France

## Abstract

**Background:**

Comparison of completely sequenced microbial genomes has revealed how fluid these genomes are. Detecting synteny blocks requires reliable methods to determining the orthologs among the whole set of homologs detected by exhaustive comparisons between each pair of completely sequenced genomes. This is a complex and difficult problem in the field of comparative genomics but will help to better understand the way prokaryotic genomes are evolving.

**Results:**

We have developed a suite of programs that automate three essential steps to study conservation of gene order, and validated them with a set of 107 bacteria and archaea that cover the majority of the prokaryotic taxonomic space. We identified the whole set of shared homologs between two or more species and computed the evolutionary distance separating each pair of homologs. We applied two strategies to extract from the set of homologs a collection of valid orthologs shared by at least two genomes. The first computes the Reciprocal Smallest Distance (RSD) using the PAM distances separating pairs of homologs. The second method groups homologs in families and reconstructs each family's evolutionary tree, distinguishing *bona fide *orthologs as well as paralogs created after the last speciation event. Although the phylogenetic tree method often succeeds where RSD fails, the reverse could occasionally be true. Accordingly, we used the data obtained with either methods or their intersection to number the orthologs that are adjacent in for each pair of genomes, the Positional Orthologous Genes (POGs), and to further study their properties. Once all these synteny blocks have been detected, we showed that POGs are subject to more evolutionary constraints than orthologs outside synteny groups, whichever the taxonomic distance separating the compared organisms.

**Conclusion:**

The suite of programs described in this paper allows a reliable detection of orthologs and is useful for evaluating gene order conservation in prokaryotes whichever their taxonomic distance. Thus, our approach will make easy the rapid identification of POGS in the next few years as we are expecting to be inundated with thousands of completely sequenced microbial genomes.

## Background

Comparison of completely sequenced genomes belonging to a wide variety of prokaryotic species has revealed how fluid these genomes are (for a review, see [1]). For instance, synteny is rapidly lost [2] as a consequence of incessant gene flows inside a genome and between species. Moreover, there is frequent gain and loss of genes, even between various strains of the same species or species belonging to the same genus (see, for example, [3]). However, a small proportion of orthologous genes, hereafter called Positional Orthologous Genes (POGs), conserve their local order even between distantly related species [4,5]. Such observations gave birth to the concept of genomic context [6,7]. Accordingly, this rare conservation of gene order has been interpreted as the signature of functional relationships between the products of these stably associated genes [8-14].

Detecting these stable islands in a sea of moving genes is a complex and difficult problem in the field of comparative genomics. The potential pitfalls increase with the deluge of newly published genome sequences. Several groups have described these difficulties, the primary one being reliably determining the orthologs among the whole set of homologs detected by exhaustive comparisons between each pair of completely sequenced genomes. Various strategies have been applied in the past to detect orthologs. The most popular one has been the so-called bidirectional best hit method. It is based on BLAST reciprocal-best-hits (RBH) between either a pair [12,15] or a triplet of genomes [16].

However, it has been shown that BLAST searches often return as the highest scoring hit a protein that is *not *the nearest phylogenetic neighbour of the query sequence [17]. As a consequence, it has been repeatedly emphasized that actual orthologs could be missed, including when using RBH approaches (see for instance [18-20] and references inside). According to Fulton et al. [20] roughly 1 in 10 RBH-predicted rat-mouse orthologs are false positives (predominantly paralogs) and about 1 in 20 RBH-predicted orthologs for two *Pseudomonas *species are probably similarly incorrect. Note however that there is presently no direct experimental validation of the effects of the Koski-Golding demonstration on the prediction accuracy of BLAST reciprocal-best-hits (or even one-way best matches) approaches.

To alleviate these problems in detection of orthologs, it seems sounder to use more theoretically consistent approaches. Wall and coworkers [18,21] have proposed a new method called RSD (reciprocal smallest distance) that is based on estimates of evolutionary distance rather than BLAST scores. Other approaches based on phylogeny criteria have been proposed for eukaryotic genomes [22-24], and more recently (as this paper was submitted) for prokaryotic data [24].

This work used two alternative and complementary approaches to reliably determine orthologs from the set of homologous genes detected after an exhaustive genomic comparison. We first adapted the RSD method [18] to our pipeline of programs for defining synteny blocks. We then introduced a new automatic approach for differentiating between orthologs and paralogs based upon automated generation of phylogenetic trees for families of homologous genes in prokaryotes. We compare the advantages and disadvantages of both the RSD and phylogeny approaches and show that, while neither method alone is always reliable, their union is giving reasonably complementary data.

This allows to recognize all *bona fide *POGs and to further identify all syntenic blocks whatever the phylogenetic distance separating the studied species. Recognizing such blocks of adjacent genes is essential to study them as ancient evolutionary units. Indeed, we further showed using statistical comparisons that some selective pressure is keeping POGs associated in a large majority of genomes. We further investigated whether POGs afford a particular mode of evolution that would be different from that of other orthologous genes which are free to move independently.

## Results

### An outline of our experimental approach

We designed a three-step experimental scheme to find the conserved syntenic blocks in a large assortment of 107 microorganisms (the complete list is given in Table 1 and Additional file 1) located at various distances from one another in the taxonomy space. In *step one*, we compared the proteome (i.e. the whole panoply of proteins encoded by a completely sequenced genome) of each organism with all other proteomes to identify the full set of homologous proteins they share. In *step two*, we developed new approaches for finding valid orthologs among all the homologs found in a comparison of each genome with all the others. *Step three *searches for a minimal conserved unit, *i.e*. a pair of POGs that are adjacent in at least two genomes. After extending these minimal units as far as possible, it becomes feasible to assess the relative number and size of synteny blocks in both nearby and distant species.

**Table 1:** List of the organisms (sorted by their taxonomy) used to compare gene order and to identify all orthologs

**Abbv**	**Domain**	**Species name**
Aerpe	Archaea	*Aeropyrum pernix*
Sulso	Archaea	*Sulfolobus solfataricus*
Pyrae	Archaea	*Pyrobaculum aerophilum*
Arcfu	Archaea	*Archaeoglobus fulgidus*
Halma	Archaea	*Haloarcula marismortui*
Halob	Archaea	*Halobacterium sp*
Metth	Archaea	*Methanothermobacter thermautotrophicus*
Metja	Archaea	*Methanocaldococcus jannaschii*
Metac	Archaea	*Methanosarcina acetivorans*
Metka	Archaea	*Methanopyrus kandleri*
Theko	Archaea	*Thermococcus kodakarensis*
Pyrab	Archaea	*Pyrococcus abyssi*
Picto	Archaea	*Picrophilus torridus*
Theac	Archaea	*Thermoplasma acidophilum*
Naneq	Archaea	*Nanoarchaeum equitans*

Symth	Bacteria	*Symbiobacterium thermophilum*
Trowh	Bacteria	*Tropheryma whipplei*
Corgl	Bacteria	*Corynebacterium glutamicum*
Leixy	Bacteria	*Leifsonia xyli*
Mycle	Bacteria	*Mycobacterium leprae*
Myctu	Bacteria	*Mycobacterium tuberculosis*
Strco	Bacteria	*Streptomyces coelicolor*
Biflo	Bacteria	*Bifidobacterium longum*
Aquae	Bacteria	*Aquifex aeolicus*
Bacth	Bacteria	*Bacteroides thetaiotaomicron*
Porgi	Bacteria	*Porphyromonas gingivalis*
Chlte	Bacteria	*Chlorobium tepidum*
Chlmu	Bacteria	*Chlamydia muridarum*
Chltr	Bacteria	*Chlamydia trachomatis*
Chlpn	Bacteria	*Chlamydophila pneumoniae*
Parac	Bacteria	*Parachlamydia species*
Dehet	Bacteria	*Dehalococcoides ethenogenes*
Glovi	Bacteria	*Gloeobacter violaceus*
Synec	Bacteria	*Synechocystis species*
Theel	Bacteria	*Thermosynechococcus elongatus*
Nosto	Bacteria	*Nostoc species*
Proma	Bacteria	*Prochlorococcus marinus*
PromM	Bacteria	*Prochlorococcus marinus*
Deira	Bacteria	*Deinococcus radiodurans*
Theth	Bacteria	*Thermus thermophilus*
Bacha	Bacteria	*Bacillus halodurans*
Bacsu	Bacteria	*Bacillus subtilis*
Oceih	Bacteria	*Oceanobacillus iheyensis*
Lisin	Bacteria	*Listeria innocua*
Staau	Bacteria	*Staphylococcus aureus*
Entfa	Bacteria	*Enterococcus faecalis*
Lacla	Bacteria	*Lactococcus lactis*
Strpn	Bacteria	*Streptococcus pneumoniae*
Strpy	Bacteria	*Streptococcus pyogenes*
Cloac	Bacteria	*Clostridium acetobutylicum*
Clote	Bacteria	*Clostridium tetani*
Thete	Bacteria	*Thermoanaerobacter tengcongensis*
Oniye	Bacteria	*Phytoplasma asteris*
Mycge	Bacteria	*Mycoplasma genitalium*
Mycpe	Bacteria	*Mycoplasma penetrans*
Mycpn	Bacteria	*Mycoplasma pneumoniae*
Mycpu	Bacteria	*Mycoplasma pulmonis*

Ureur	Bacteria	*Ureaplasma parvum*
Mesfl	Bacteria	*Mesoplasma florum*
Fusnu	Bacteria	*Fusobacterium nucleatum*
Pirel	Bacteria	*Rhodopirellula baltica*
Caucr	Bacteria	*Caulobacter crescentus*
Braja	Bacteria	*Bradyrhizobium japonicum*
Rhopa	Bacteria	*Rhodopseudomonas palustris*
Brume	Bacteria	*Brucella melitensis*
Meslo	Bacteria	*Mesorhizobium loti*
Agrtu	Bacteria	*Agrobacterium tumefaciens*
Sinme	Bacteria	*Sinorhizobium meliloti*
Anama	Bacteria	*Anaplasma marginale*
Ricco	Bacteria	*Rickettsia conorii*
Ricpr	Bacteria	*Rickettsia prowazekii*
Wolba	Bacteria	*Wolbachia pipientis*
Zymmo	Bacteria	*Zymomonas mobilis*
Borbr	Bacteria	*Bordetella bronchiseptica*
Ralso	Bacteria	*Ralstonia solanacearum*
Neime	Bacteria	*Neisseria meningitidis*
Niteu	Bacteria	*Nitrosomonas europaea*
Bdeba	Bacteria	*Bdellovibrio bacteriovorus*
Desps	Bacteria	*Desulfotalea psychrophila*
Desvu	Bacteria	*Desulfovibrio vulgaris*
Geosu	Bacteria	*Geobacter sulfurreducens*
Sheon	Bacteria	*Shewanella oneidensis*
Blofl	Bacteria	*Blochmannia floridanus*
Buchn	Bacteria	*Buchnera aphidicola*
Escco	Bacteria	*Escherichia coli*
Salen	Bacteria	*Salmonella enterica*
Wiggl	Bacteria	*Wigglesworthia glossinidia*
Yerpe	Bacteria	*Yersinia pestis*
Coxbu	Bacteria	*Coxiella burnetii*
Haein	Bacteria	*Haemophilus influenzae*
Pasmu	Bacteria	*Pasteurella multocida*
Acine	Bacteria	*Acinetobacter species*
Pseae	Bacteria	*Pseudomonas aeruginosa*
Fratu	Bacteria	*Francisella tularensis*
Vibch	Bacteria	*Vibrio cholerae*
Vibpa	Bacteria	*Vibrio parahaemolyticus*
Xanax	Bacteria	*Xanthomonas axonopodis*
Xylfa	Bacteria	*Xylella fastidiosa*
Camje	Bacteria	*Campylobacter jejuni*
HelpJ	Bacteria	*Helicobacter pylori*
Helpy	Bacteria	*Helicobacter pylori*
Wolsu	Bacteria	*Wolinella succinogenes*
Lepin	Bacteria	*Leptospira interrogans*
Borbu	Bacteria	*Borrelia burgdorferi*
Trede	Bacteria	*Treponema denticola*
Trepa	Bacteria	*Treponema pallidum*
Thema	Bacteria	*Thermotoga maritima*

### Collecting homologous genes in 107 microorganisms

In this first step, we compared each organism's proteome with those of all the others to identify the full set of homologous proteins they share. Accordingly, we made 5671 comparisons between every pairing of the whole set of 107 prokaryotic species (93 bacteria and 14 archaea) listed in Table 1. To ensure that the search for homologs was both exhaustive and exact, regardless of the taxonomic distances separating the compared species, we used the DARWIN AllAll program [25,26] to compare each pair of proteomes. As previously shown [27], the maximum likelihood approach used by the DARWIN programs efficiently detects *in one step *all segments of homology, including those between evolutionarily-distant proteins [28]. These features are crucial in work such as this that compares a large number of genomes separated by a wide range of phylogenetic distances.

An additional advantage of the DARWIN programs is that the evolutionary distance between similar proteins in species with a common ancestor uses the well-established PAM units, a PAM unit being defined as the number of *accepted *point mutations per 100 residues separating two sequences [29,30]. The frequency with which any particular pair of (mutated) amino acids occurs at a given position in two properly-aligned homologous proteins can be used as a PAM score to evaluate the evolutionary distance separating the two proteins. Accordingly, as previously described [27] and detailed in Methods, we applied the rules proposed by Altschul [31] to define how significant sequence similarities are. We required that the PAM distance separating a pair of homologs be less than 250 units and that each segment of homology have a minimal size of 80 residues. Moreover, in this search for homologs belonging to syntenic blocks, we further required that the pair of aligned amino acid sequences extend at least 80% of the length of the shorter matching protein.

The AllAll homolog search compared each of the 295,608 proteins to the whole set encoded by the 107 prokaryotic genomes (Table 1). We found that the vast majority (272,472) of these proteins have at least one homolog in another microorganism, probably because many compared species have at least one (often very-) close taxonomic relative in the data set (e.g. *Escherichia coli *and *Salmonella enterica*).

### Reliable determination of orthologous genes

In this *second step*, we tried to recognize a reliable set of orthologs among the full set of 272,472 homologs the first step had found. As explained in the introduction, this is not a trivial task; so we compared the results of two alternative strategies to avoid the pitfalls of applying each method independently.

#### (i) Identifying orthologs using a Reciprocal Smallest Distance (RSD) approach

We used a reciprocal evolutionary distance to avoid the risks of errors inherent in similar previous approaches explained in the introduction. We took advantage of specific properties of the DARWIN AllAll program [25,26] that set it apart from BLAST algorithm. Also, the AllAll program calculates the PAM evolutionary distances separating homologous genes, retaining the shortest distance found for each analyzed pair of proteins. These features made it trivial to determine RSD ortholog pairs in a comparison of two proteomes, using a strategy similar to that of Wall and coworkers [18,21]. The RSD ortholog pair between protein *a *encoded by genome G_A _and protein *b *encoded by genome G_B_, was the one for which the PAM distance separating *a *from *b *was smaller than that separating *a *from any other protein encoded by G_B _and *b *from any other protein encoded by G_A_.

This RSD approach produced a list of 204,792 orthologs amounting to 69% of the proteins encoded by the 107 genomes studied in this paper. These 204,792 orthologs form a total of 2,332,248 pairs (Table 2).

**Table 2:** Identifying sets of synteny blocks using two different approaches to detect orthologs after comparing the proteomes of 107 microbial species

Detection method	Number of pairs of orthologs	Blocks of two adjacent orthologs	Syntenic blocks	
			
			Number	Mean size
RSD	2,332,248	290,108	182,289	2.59 genes
Phylogeny	2,255,324	302,468	186,744	2.62 genes
Union	3,014,995	377,256	235,519	2.60 genes
Intersection	1,572,577	226,799	149,058	2.62 genes

However, we found that this one-to-one approach would miss a significant number of orthologous relationships when gene duplications arose after a recent speciation event. Therefore, we developed a second approach based on phylogenetic analyses that detects these so-called in-paralogs [32].

#### (ii) Identifying orthologs by a phylogenetic approach

This second approach analyses the evolutionary features of the detected homologous proteins after grouping them in families. A transitive approach based on a graph algorithm for determining connected components linked the 272,472 homologous proteins into 12,719 families. Unfortunately, we observed that a few of these families were enormous, heterogeneous groupings that, in a significant number of cases, consisted of two or more groups of highly-connected proteins linked by a few probably-spurious edges. Although many of these bridges are indicating a biologically meaningful paralogy, they appear detrimental to our search for consistent and valid families required to identify genuine orthologous relationships. To avoid any disadvantageous groupings of paralogous families and to reduce the size of too-large and heterogeneous families, we further applied a graph algorithm for bridge detection, and broke many unwanted bridges (see Methods). Applying this strategy to the 12,719 transitive families redistributed all the homologous genes into 13,139 better defined families.

**Figure 1:**
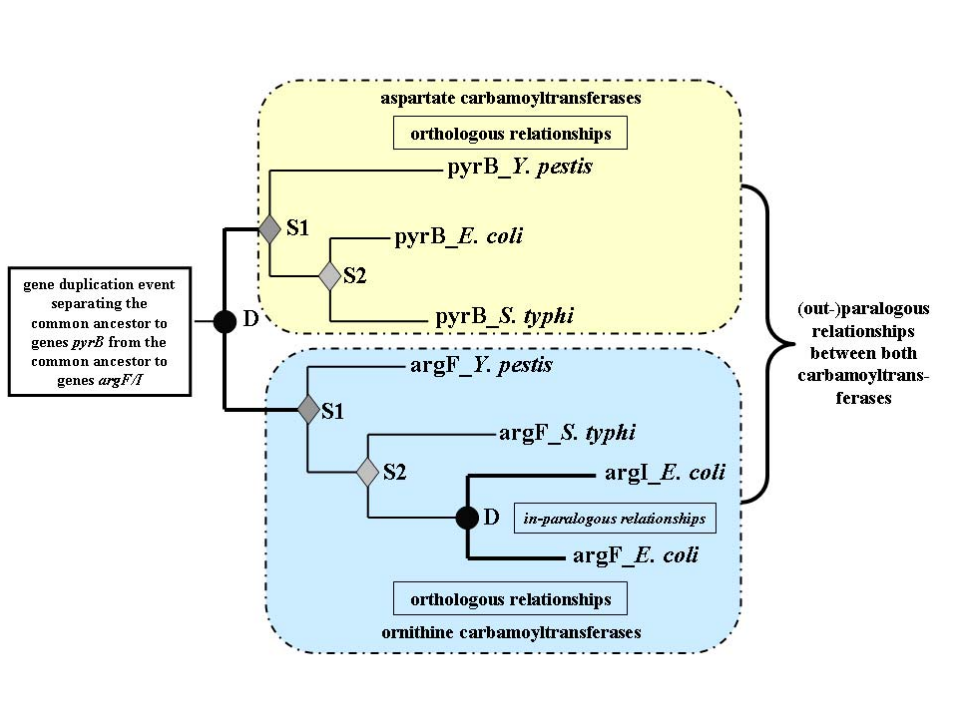
**Differentiating orthologous and paralogous relationships in phylogenetic trees**. This simplified tree is adapted from a previous work on the evolution of carbamoyltranserases [60]. The nodes corresponding to the respective events of ancestral gene duplication D (creating paralogous groups of carbamoyltransferases) and speciation S1 and S2 are identified by a black circle and a gray diamond, respectively. The presence of in-paralogs is shown by thick branches leading to leaves corresponding to genes belonging to the same species.

**Figure 2:**
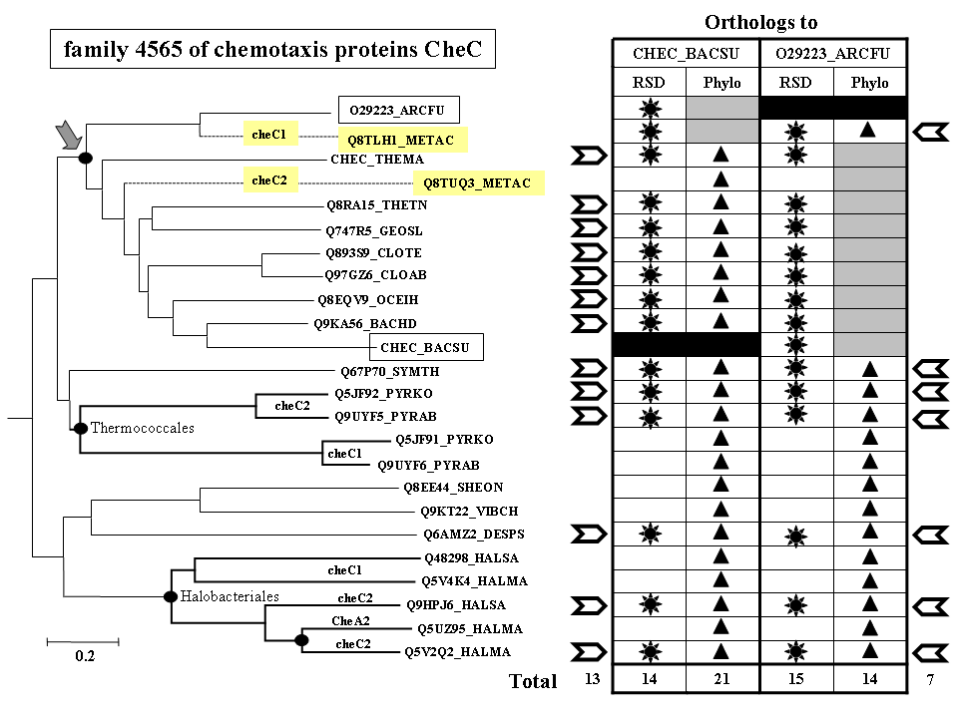
**Phylogenetic tree showing pros and cons of both ortholog detection methods**. We used PhyML [59] to reconstruct a maximum likelihood tree for family 4565 that groups chemotaxis proteins CheC. The table on the right summarizes the data obtained when listing the orthologs of the *Archaeoglobus fulgidus *(O29223_ARCFU) and the *Bacillus subtilis *(CHEC_BACSU) sequences respectively. Orthologs found by the RSD approach are indicated by a star, those detected with the phylogeny approach by black triangles, and those found by both methods with a chevron. All nodes are supposed to correspond to speciation events except those labeled with a black dot, which are assumed to be due to gene duplication events.

After this step, one extremely large protein "family" remained. This heterogeneous cluster contained 107,219 members that are mainly hydrophobic proteins such as transporters and other membrane proteins, including many (20,607) proteins with unknown function. Such a huge gathering of disparate proteins is biologically meaningless. Moreover, it was clearly worthless to analyze it by a tree approach due to its size and complexity (see below, Figs. 1 and 2). Therefore, we applied the MCL algorithm [33,34] to break up this huge and heterogeneous cluster.

This left 190,770 homologous proteins that are members of 21,371 families: 9,405 pairs of proteins (families of size 2) and all the homologous proteins belonging to each of the remaining 11,964 families of size 3 or larger – including the largest one that contains 350 proteins. After reconstructing a phylogenetic tree for each family, we applied an *ad hoc *algorithm (see Methods and Additional file 2) to identify the orthologous relationships within each phylogenetic tree, as shown in Fig. 1 and detailed in Methods. As it has been repeteadly shown, there is no prokaryotic species tree that is recognized as a consensus. Accordingly, we used an approach that does not require such a model tree. Briefly, the *treeortho *algorithm analyses the leaves of a midpoint-rooted tree to determining for each node – whichever its deepness – whether its descendants arose from a gene duplication (sharing several sequences in the same species) or a speciation event (no common species). For instance, the deepest node of the simplified tree in Fig. 1 is viewed as a duplication event (D, black circle) because the two branches stemming from this node display leaves that correspond to the same set of species. The descendents of the immediate next nodes being in separate species, these nodes identify speciation events (S, gray diamond).

Probing all the family trees that we could analyze by this method recovered a total of 190,770 proteins that have an ortholog in at least one another species. In total, this phylogeny approach yielded a total of 2,255,324 orthologous pairs (Table 2).

#### (iii) Comparing the RSD and phylogenetic approaches

Table 2 summarizes the results obtained with the two approaches. Although the total number of orthologous pairs obtained by both methods is similar, it is striking that they have two thirds of their predictions in common, namely 67% of the RSD orthologs and 69% of the phylogeny ones, respectively.

Fig. 2 shows a tree which illustrates the pros and cons of both approaches. Family 4565 groups chemotaxis proteins CheC in different Euryarchaeota, Proteobacteria, Firmicutes, one Actinobacteria and *Thermotoga maritima*. Consider first the search for the orthologs of the *B. subtilis *CheC protein (CHEC_BACSU). The RSD approach finds only 14 orthologs (indicated with a star in the table), discarding the paralogs present in *Thermococcus kodakarensis*, *Pyrococcus abyssi *and the halophilic archaea *Haloarcula *and *Halobacterium *(thick branches). The phylogeny approach detects all these in-paralogs, going up to 21 retained proteins (indicated with a triangle). Thus, in this case the phylogeny approach appears to be far more efficient, as expected.

Let's take now the search for the orthologs of the CheC protein in *Archaeoglobus fulgidus *(O29223_ARCFU). Here the RSD approach finds 15 orthologs (indicated with a star), and the phylogeny approach 14 (triangles) but only 7 (indicated with a chevron) are found by both approaches. By definition, seven paralogs present in the different archaeal species (e.g. Q5V4K4_HALMA, the product of the *cheC1 *gene in *Halobacterium marismortui*) were excluded by the RSD approach but included in the harvest of the phylogeny approach after identification of the nodes corresponding to a duplication event (black dots) and those corresponding to a speciation event. However, the presence in the dataset of two different sequences (Q8TLH1_METAC, the product of the *cheC1 *gene and Q8TUQ3_METAC, the product of the *cheC2 *gene) for the same organism (*M. acetivorans*) led the *treeortho *algorithm to identify as a gene duplication node (arrow) the separation between the two subtrees containing the respective *M. acetivorans *sequences. As a consequence, all the nine descendents of the ancestral copy that led to the *M. acetivorans cheC2 *sequence were excluded from the phylogenetic tally (indicated in gray in the table of Fig. 2) as corresponding to a paralogy event. These descendants are found by the RSD approach because there are only one remaining copy in ARCFU (CheC1) and one remaining copy (CheC2) in each of the seven different bacteria branching in the CheC2_METAC subtree. Note that the ancestral *cheC *duplication appears rather ancient as shown in a tree containing all archaeal cheC sequences and rooted with a few bacterial homologs (see Additional file 3). The two copies have been separated by at least three speciation events, the *cheC2 *copy having highly diverged (note its branch length) and appearing now more similar to a group of bacterial *cheC*. This large divergence is further underlined by the respective gene context of both copies of *cheC*. Although, there was an ancestral duplication of the whole operon, we found that the adjacency *cheD1 *– *cheC2 *is conserved in the various phylogenetically close bacteria (adjacency *cheD *– *cheC *in *Thermus thermophilus*, *Geobacter sulfurreducens*, *Oceanobacillus iheyensis*, *Bacillus halodurans*, and *B. subtilis*). On the contrary, gene *cheC1 *is found adjacent to *cheA1 *in *M. acetivorans *as it is in the other Euryarchaeota.

Such correct algorithmic interpretations that lead to reject RSD orthologs as being actual paralogs probably explain many of the cases of sequences that are discarded by phylogeny but recovered by RSD. Such instances could not occur in the 6278 low-complexity trees that are made uniquely of orthologs, without paralogs, but may happen when analysing the 1011 trees of medium complexity containing only orthologs and in-paralogs and the 4675 trees of high complexity that contain in- and out-paralogs, as well as orthologs.

#### (iv) Combining data obtained with the RSD and phylogenetic approaches

Both approaches, thus, yield incomplete data. The union of both result sets provides a total of 3,014,995 pairs of orthologs (Table 2). Because of the potential for confusion from the false positives returned by both methods, it must be noted that their intersection left 1,572,577 pairs of orthologs that are endowed with a high degree of confidence that they are genuine. However, such a stringent filtering removes all in-paralogs.

### Identifying pairs of adjacent orthologous genes

Given the lists of *bona fide *orthologs, our *third step *is to determine how many form reciprocal pairs of strictly adjacent genes in at least two genomes. We found that the most efficient and rapid way to collect all these pairs was the following strategy. We stored all the data obtained from the comparison of the 5671 pairs of genomes in a relational database SynteBase (see Additional file 3) and we designed a specific SQL request (see Methods) to query SynteBase to detect the quadruplets of proteins containing two pairs of orthologs that are encoded by genes which are adjacent in both genomes. Table 2 shows that we detected 290,108 synteny blocks of size 2 with the RSD method and 302,468 ones with the phylogenetic analysis. The slight excess of synteny blocks found by the phylogeny approach is probably due to the detection of remote homologs in families that could not be found in RSD pairs due to our initial alignment threshold. This is most probably the case, for instance, of the CheC sequences encoded by *Shewanella oneidensis *(Q8EE44_SHEON) and *Vibrio cholerae *(Q9KT22_VIBCH) which are not detected by RSD but found by tree analysis as orthologs of *B. subtilis *CheC (Fig. 2). Table 2 further shows that we get more pairs of orthologs with RSD but fewer blocks of synteny, underlining that some RSD-orthologs are most probably ancient paralogs (pseudoorthologs). Summing all data obtained with both methods provides a total of 377,256 blocks of two adjacent orthologs (Table 2).

Finally, we attempted to extend the detected neighbourhood relationships to larger syntenic blocks. The quadruplets, stored in SynteBase, were analyzed with *synblock*, an algorithm we designed to map the synteny blocks of size 2 and to merge those that share a common pair of orthologous genes. Note that, in this work where we aim to find out the conserved blocks of genes that reflect the ancestral gene order, we required strict gene adjacency, forbidding any insertion in a synteny block of a gene which would be unique to one of the pairs of compared genomes.

Table 2 shows that the respective numbers of larger blocks were 182,289 for the RSD approach and 186,744 for the phylogenetic analysis. After summing data from both methods, we got a total of 235,519 synteny blocks. Note that the average size of these blocks is 2.60 genes per block (2.59 for the RSD ones and 2.62 for the phylogeny ones, respectively). These data confirm how fluid prokaryotic genomes are when they are studied at any taxonomic distance.

We further used these data entered into SynteBase to make some statistical studies on the respective level of conservation of neighbourhood and other evolutionary features linked to gene order.

### Assessing the evolution rates of positional orthologous genes

#### Measuring the distribution of the PAM distances in the taxonomic space

**Figure 3:**
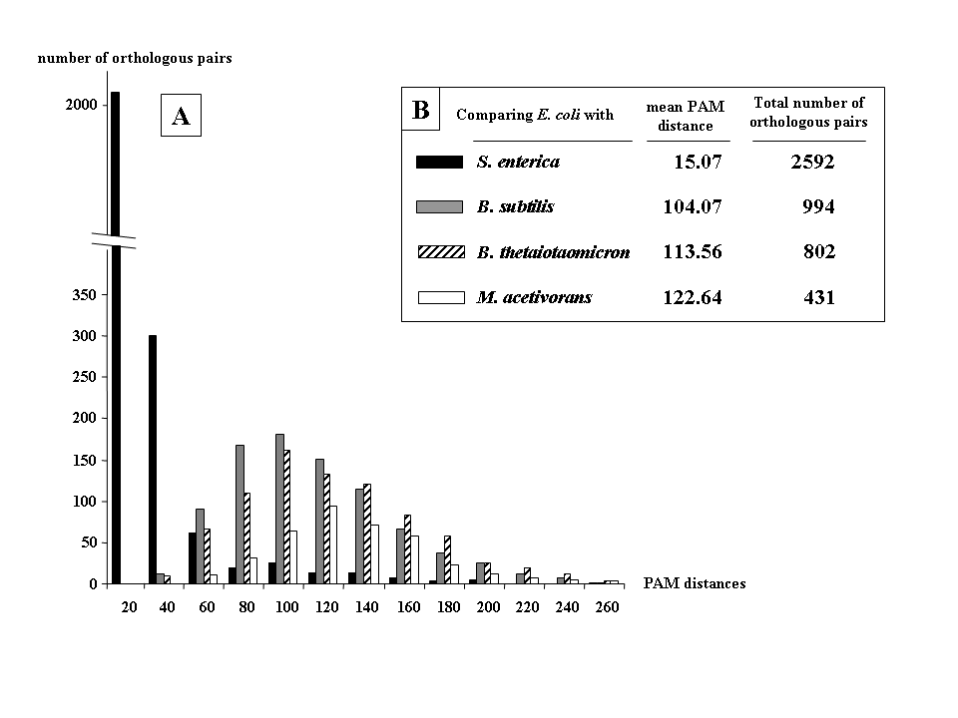
**Comparing the distribution of PAM distances separating pairs of orthologs shared between the proteomes of *E. coli *and four other organisms**. Panel A (upper part) shows the respective distributions of the PAM distances for all pairs of orthologs shared by *E. coli *and *S. enterica*, *B. subtilis*, *B. thetaiotaomicron *and *M. acetivorans*, respectively. The color code is given in box B, which also compares the respective mean of each PAM distance distribution and the total number of orthologs shared by each pair of genomes analyzed in A.

The PAM distance that separates two orthologs in various pairs of genomes was used to compare the rate of evolution of different classes of orthologs with different respects. First, Fig. 3A shows the distribution of the PAM distances separating orthologous pairs when comparing *E. coli *with closely related Enterobacteriaceae *S. enterica*, the Firmicute *B. subtilis*, the member of the Bacteroidetes/Chlorobi group *Bacteroides thetaiotaomicron *and the archaeon *M. acetivorans*. As expected, this distribution is very dependent of the taxonomic proximity between species. This is confirmed in Fig. 3B and Table 3 that display the taxonomic distributions of the mean of the PAM distances separating pairs of orthologs and of the total number of orthologous pairs in the different genomic comparisons.

**Table 3:** The mean PAM distance separating two orthologs and the average size (number of genes) of synteny blocks are dependent on the taxonomic distance separating the two genomes that have been compared at the level of their genetic context

Hierarchy rank	**Species 1**	Species 2			Mean PAM distance	Synteny block	
	(***E. coli***^1^) Taxonomy	Species name	Taxonomy	Proteome size		Mean size	Longest size

Family	Enterobacteriaceae	*S. enterica*	Enterobacteriaceae	4318	15.07	3.36	20
Order	Enterobacteriales	*V. cholerae*	Vibrionales	3835	61.89	2.92	10
	Enterobacteriales	*P. aeruginosa*	Pseudomonadales	5567	79.64	2.94	12
Class	Gamma proteobacteria	*M. loti*	Alpha proteobacteria	6746	103.05	2.47	9
Phylum	Proteobacteria	*B. subtilis*	Firmicutes	4112	104.07	2.41	9
	Proteobacteria	*M. tuberculosis*	Actinobacteria	3995	109.58	2.31	6
	Proteobacteria	*C. tepidum*	Bacteroidetes/Chlorobi	2252	105.03	2.78	9
	Proteobacteria	*R. baltica*	Planctomycetes	7325	112.53	2.16	8
Domain	Bacteria	*M. acetivorans*	Archaea (Euryarchaeota)	4540	122.64	2.13	3
	Bacteria	*S. solfataricus*	Archaea (Crenarchaeota)	2977	132.63	2.05	3

^1 ^proteome size of *E. coli *K12: 4279

Table 3 summarizes the data obtained when comparing the model organism *E. coli *with various bacteria and archaea separated from it by increasing ranks of the taxonomy hierarchy such as family, order, class, phylum and Domain, respectively. Such comparisons show that the mean PAM distance separating the orthologs of *E. coli *from those of other organisms increases rapidly when moving far away in the taxonomic space from family to class ranks. Although this was expected, we observed that these separating distances were not unbounded, reaching a plateau value of around 120 PAM units when comparing the different bacterial phyla and around 140 PAM units when comparing Domains Bacteria and Archaea. Table 3 further shows that the size of conserved synteny blocks also depends on the phylogenetic (taxonomic) distance between species. Indeed, the mean maximum synteny block size is nearly 3.5 genes when comparing two closely related bacteria such as the Enterobacteriaceae *E. coli *and *Salmonella enterica*, but goes down to the minimal size of 2 when comparing a bacterium (*E. coli*) and an archaeon (*M. acetivorans*), although these genomes encode a similar range of proteins. Likewise, the longest synteny block is only 3 when comparing domains Bacteria and Archaea, but goes up to around 9 when comparing organisms of the same class or phylum, and around 11 within the same order. The longest block for the two studied Enterobacteriaceae contains 20 adjacent genes. The record up to now is 30 adjacent genes for the pair *B. subtilis *– *B. halodurans *(see Additional file 5).

#### Measuring the evolution rates of the different classes of orthologous genes

**Figure 4:**
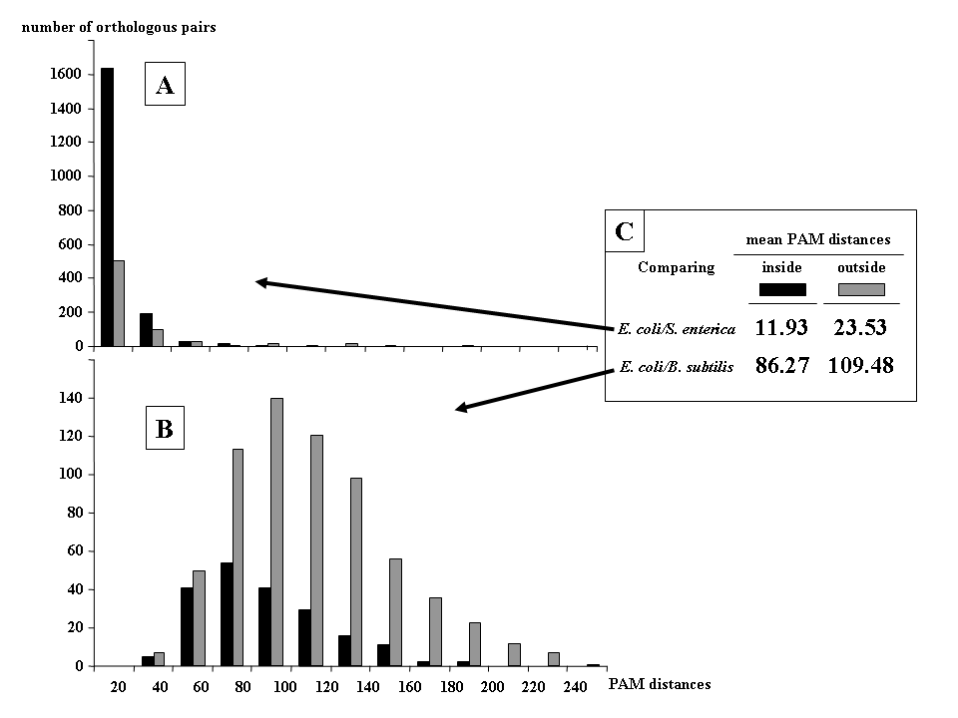
**PAM distance distributions for orthologs pairs inside and outside synteny blocks**. Part A (upper) compares all pairs of orthologs shared by closely related enterobacteria *E. coli *and *S. enterica*. Part B compares a more distant pair of genomes, *E. coli *and *B. subtilis*. The color code is given in box C, which also compares the respective mean of each distribution of PAM distances and the total number of orthologs shared by each pair of genomes analyzed in A and B.

Table 3 shows that the evolutionary distance separating POGs levels off at less than 150 PAM units, even for extremely distant species. This suggests that some strong constraint is exerted on the evolution of these peculiar genes. To check this evolutionary model, we further compared the PAM distance distributions for orthologous genes located either inside synteny blocks (the so-called POGs) or outside these blocks. Fig. 4 shows that the PAM distances appear to always be less inside than outside the synteny blocks. This difference (summarized in Fig. 4C) appears to be independent of the taxonomic distance separating species, since we observe it as well for the *E. coli*/*S. enterica *pair (Fig. 4A) as the *E. coli*/*B. subtilis *pair (Fig. 4B). This is confirmed by Table 4, which displays the mean PAM distances between POGs and non-POGs for the set of species already analyzed in Fig. 3 and Table 3. In all cases, the PAM distances are shorter inside the blocks than outside, even though there are fewer and fewer of these synteny blocks as the taxonomic distance separating species increases.

**Table 4:** Comparing the means of Pam distances and the total number of orthologs located inside or outside synteny blocks and shared by four pair of genomes

*E. coli *proteome compared with	Mean PAM distance		Number of orthologous pairs		
	
	Inside	Outside	Total	% inside	% outside
*S. enterica*	11.93	23.53	2592	27	73
*B. subtilis*	86.27	109.48	994	23	77
*B. thetaiotaomicron*	109.14	114.42	802	16	84
*M. acetivorans*	113.01	124.53	431	14	86

To check if the observed difference between the PAM distances separating orthologous genes located inside and outside synteny blocks (Fig. 4 and Table 4) is statistically significant for any pair of species, we further tested the whole set of compared genomes, using a bootstrap sampling approach as detailed in Methods. The null hypothesis (H_0_) assumes that proteins encoded by POGs evolve at the same evolutionary rate as those encoded by orthologous genes located outside the synteny blocks. The alternative hypothesis (H_1_) assumes that the proteins encoded by orthologous genes located inside synteny blocks evolve more slowly than the proteins encoded by orthologous genes located outside synteny blocks.

**Figure 5:**
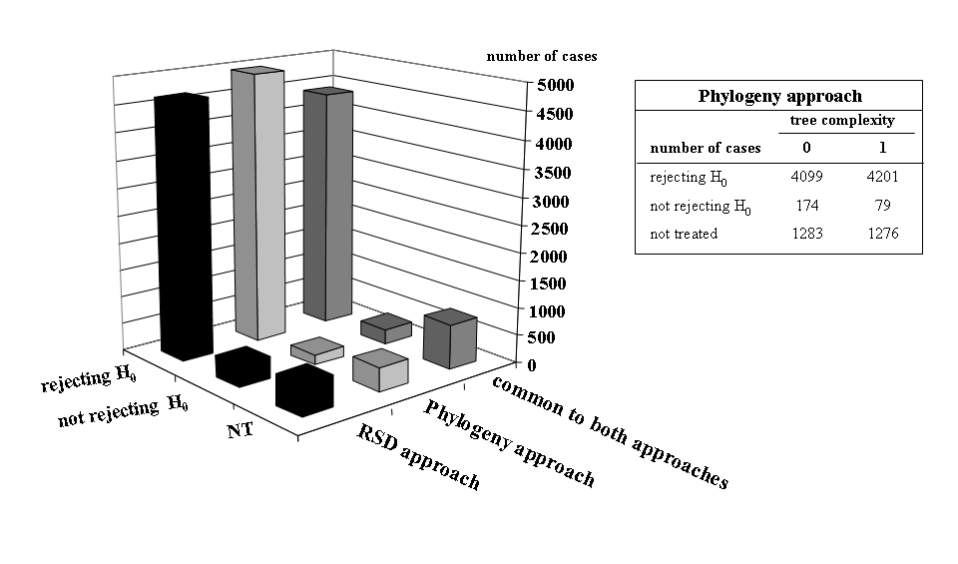
**Bootstrap analysis of the distribution of PAM distances separating pairs of orthologs located inside and outside synteny blocks**. The number of cases rejecting and not rejecting hypothesis H_0_, and those corresponding to cases too small to be included in this statistical test are shown for each method (RSD and phylogeny) and their intersection. The inset table details the data for trees of complexity 0 (orthologs only) or 1 (orthologs and in-paralogs).

To validate this study, we first used the phylogenetic approach, taking into account orthologs contained in trees of various complexities defined as follows. We analyzed trees made up uniquely of orthologs (complexity 0), and trees containing only orthologs and in-paralogs (complexity 1). We then did similar computations for the 107 genomes with the entire dataset obtained using the RSD ortholog detection method. Fig. 5 shows that in both RSD and phylogeny approaches, the overwhelming majority of the statistical tests reject the null hypothesis H_0 _in favour of H_1_. We found that the remaining, untested cases (NT) correspond to a few comparisons that are too small to be safely used in this bootstrap test. This is the case, for example, of all comparisons (1% of the total) involving *Nanoarchaea equitans*, an archaeon with a very small genome that is also very distant from all other species, including the other archaea.

We conclude that there is a universal trend in the evolution of prokaryotic genomes: genes located in regions with preserved gene order (POGs) evolve more slowly than genes found outside.

## Discussion

As soon as several prokaryotic genome sequences were completed, it became clear that very few of their genes had retained their local ordering through evolution. This observation was reinforced as more and more genomes belonging to a wide spectrum of organisms – from very close to very distantly-related – were published. This led to the concept of gene (genomic) context [7-14,35]. As we prepare to be inundated by thousands of completely sequenced genomes in the next few years [36], it becomes urgent to design a strategy for quickly evaluating the conservation of gene order in newly-published genomes, comparing them to all those already analyzed.

This paper describes a suite of programs (summarized in Table 5) that automate all the essential steps for evaluating gene order conservation in prokaryotes, as well as presenting some results from their initial application. After identifying in step 1 (Table 5) the whole set of homologs shared by 107 prokaryotic species (Table 1), we designed two independent strategies (steps 2a to 2e) to extract from this set the collection of orthologs that are shared by at least two genomes. In a third step (3a and 3b), we located the positional orthologous genes (POGs) in this collection, and we studied the specific properties that distinguish them from other orthologs.

**Table 5:** A suite of programs to identify synteny blocks

Step	Step designation	Tool	Reference
1	Identifying homologs	Darwin AllAll	Gonnet et al., 1992
2a	Identifying orthologs by RSD	Perl script *rsd_ortho*	FL, this work
2b	Clustering homologs	Perl script *famtrans*	FL, this work
2c	Breaking bridges	Graph algorithm (Perl graph library) *cutbridge*	FL, this work
2d	Extracting significant clusters	MCL algorithm	van Dongen, 2000
2e	Identifying orthologs in evolutionary trees	Perl script *tree_ortho*	FL, this work
3a	Identifying pairs of adjacent orthologous genes	Querying PostgreSQL database SynteBase	FL, this work
3b	Finding out synteny blocks	Perl script *synblock*	FL, this work

### Correctly identifying positional orthologs

This is a crucial step, because mixing paralogs and orthologs can lead to errors in predicting ortholog pairs that seriously compromise studies of the mode of evolution of genes and genomes. This is especially true when studying prokaryotes. We already showed [27,28,37] that many gene families stem from ancestral gene duplication events that occurred well before the divergence of present-day species. Therefore, orthologs are often more similar between closely related species, allowing them to be distinguished from paralogs. However, when we compare genomes belonging to distant species, the difference in percent identity between orthologs and paralogs falls rapidly (see [28] for a detailed analysis), and the risk of confusing errors quickly increases.

Although there is a long history of research on identifying orthologs, this is an extremely active research area at the moment (for a review, see [38]). Numerous papers appeared within months, each one claiming to present the "best method" (see, for instance, [5,19-21,39-45]). A consensus does seem to be emerging about the superiority of approaches based on evolutionary studies over best bidirectional BLAST hits (see, however, the recent work of Duthil et al. [46] about the specific case of fungi). Anyhow, the fact that so many "best methods" were published in a single year clearly underlines that the field is immature.

Because no single approach is superior in all cases, our work applies two alternative and complementary techniques, Reciprocal Smallest Distance (RSD) and phylogenetic approaches. Both approaches are based on well-established PAM evolutionary distances, computed by a maximum likelihood approach [25,26]. Using these PAM distances allows directly determining the ortholog pairs separated by RSD, comparing each homolog with all others by means similar to the method already proposed by Wall et al. [18]. In our 107 genomes, this method identified 204,792 orthologs (69% of the total compared proteins) forming 2,332,248 pairs.

We expected that the phylogeny approach would systematically increase these figures; since it should collect not only all pairs of orthologs found by RSD but also so-called in-paralogs [32] the RSD approach misses. In fact, this does happen in many cases. For example, as Fig. 2 shows for orthologs of the *B.subtilis *chemotaxis CheC protein, we increase the number from 14 found using the RSD approach to 21 with the phylogeny approach. Unfortunately, although this phylogeny strategy was found to be quite effective, it can create unexpected difficulties when comparing many protein sequences that form some heterogeneous and very large "families". In fact, these huge agglomerates contained about the half of all found homologs. We managed to decompose many of these into more valid and much smaller families. However, this decomposition step caused the loss of a significant number of genuine pairs of orthologs.

On the other hand, phylogenetic analysis is advantageous when it rejects a significant number of homologous pairs that have been found as orthologs by the RSD approach, as being actual paralogs. This will occur for instance when a deep node is correctly viewed as due to an ancestral gene duplication event (Fig. 2). Moreover, the apparent excess of pairs of orthologs found by the RSD method (Table 2) looks suspicious; since a significant number of them are discarded when looking at the number of POGs found by this approach. Therefore, it seems that phylogeny approach is more accurate to detect *bona fide *orthologs, excluding pseudoorthologs (actual paralogs that appear to be orthologous due to differential lineage-specific gene loss) and including in-paralogs.

However, it appears presently hazardous to choose a single "best method" to identify orthologs. Our present strategy has been to combine the results of our two alternative methods. Uses of the data fall between two possible options extremes: using the union and using the intersection of their results. Where the goal is to collect a maximum of putative synteny cases in the maximum number of genomes, it is appropriate to merge the orthologs determined by both methods. Alternatively, if the goal is to work with pairs of orthologs that are highly likely to be authentic, it would be best to use only the results that are common to both approaches, but such an intersection will unfortunately remove all in-paralogs.

#### Evolutionary constraints on POGs

Based upon these identified synteny blocks and the computed (PAM) evolutionary distances separating the proteins encoded by these adjacent genes (Fig. 3), it was possible to compare the rates of evolution of the orthologous genes located inside and outside synteny blocks, respectively. As shown in Table 4 and Figs. 4 and 5, proteins encoded by genes located within synteny blocks are found to evolve at a lower rate than those encoded by genes located outside these blocks.

This observed difference appears to be consistent with the concept of genomic context in which it is assumed that neighboring genes are under a strong evolutionary pressure to maintain their adjacency. Such a pressure would exist, for example, if the proteins they encode have to interact in order to participate to the same biological process. However, in most cases it is not yet clear what the actual cause of low evolutionary rate is.

Our demonstration that POGs are more evolutionarily constrained than other orthologs that do not reside in synteny blocks confirms and extends the previous observation made by Dandekar *et al.*[8] that "the degree of sequence conservation is on average substantially higher than that in genes that do not exist as conserved genes pairs". Moreover, our demonstration agrees with both the variables and universals (namely essentiality, expression level, and connectivity) that have previously been associated with differences in protein evolutionary rates (for a recent review, see [47]). We expect that persistently adjacent genes result from several of these universals: (i) they are probably essential since they are conserved in large parts (if not all) of the taxonomic space. (ii) They will be co-transcribed (whether or not they are part of an operon) at the same level of expression. (iii) In many cases, their products will interact in the cell, requiring a significant level of connectivity between them and strict conservation of residues to permit a sufficient density of structural and functional contacts.

We performed a preliminary evaluation of these hypotheses using experimental data obtained from the model organisms *E. coli *and *B. subtilis*. Of the 204 POGs shared by these two species, 53 (26%) are essential *E. coli *genes, 130 non-essential and 21 unknown. Moreover, 102 of the shared *E. coli *and *B. subtilis *POGs (50% of the total 204) belong to the *E. coli *interactome and 47 of these are essential genes (89% of the 53 essential genes). In total, around 23% of the 204 POGs *E. coli *shares with *B. subtilis *are both essential and part of its interactome, making them what have been called *hub proteins *[48].

The different data obtained in this work suggest that blocks of adjacent POGs which display conserved co-occurence in various present-day genomes can be interpreted as ancient evolutionary units that have resisted over the years to the imperious fluidity forces. Thus, these evolutionary units could be viewed as the remnants of the skeleton of the gene order found in a common ancestor to all compared present-day microorganisms.

We further propose that POGs have a low evolutionary rate because they are constituents of functional modules as previously defined by Hartwell *et al.*[49] in a systems biology perspective (for a recent review, see [50]). Being a part of such modules would require a coordinated evolution between their sequence and expression level to participate in a collective cell function.

## Conclusion

Maintaining the order of genes whose products interact in the same cell function is clearly beneficial to organisms, for instance to allow co-expression and co-regulation. This requires the creation of clusters that have low recombination rates (according to Fisher [51]) and that the adjacency of functionally related genes is maintained by epistatic selection [52]. However, as we compare species that are distant in the taxonomy space, there is an inverse relation between taxonomic proximity and the size of the synteny blocks. This evidence agrees with Poyatos and Hurst [53] contention that gene order is forever in flux. Therefore, the exceptional instances where a stable gene order is conserved, even for a few gene pairs, implies strong selective pressure that should be reflected in high levels of co-expression of essential products such as one sees, for instance, in the exemplary case of ribosomal proteins.

## Methods

### Comparing protein sequences

The whole set of translated coding sequences (proteome) obtained for completely sequenced genomes is first imported from GenBank or EMBL databanks. A Perl script extracts the following information for each protein of each genome: a protein identification number, the species name, the gene name, the length of the protein and its sequence. The script produces output in a SGML format required to perform homology search using the DARWIN 3.0 package [25,26].

Their DARWIN AllAll program searches a protein database for homologs using a maximum likelihood approach. We have adapted this AllAll program to our own use ([27], see also [54]). It uses a two-step procedure [25,26]. First, it applies a dynamic programming algorithm [55] with a PAM 250 substitution score matrix [30]. In this first step, the introduction of gaps is strongly discouraged by penalties computed as a function of the PAM distance separating the two sequences [56]. Then, in a second step, each alignment is refined using two other tools. The first tool attempts to extend the initial alignment as far as possible using the Smith and Waterman algorithm [57]. The second tool recalculates the initial substitution score matrix using the current data set of proteins, and the process is repeated. The optimization process is monitored by computing the variance of the PAM distance. When it cannot be decreased further, the alignment is registered as the optimal one for the two proteins studied.

To detect distant homologs, we applied as already described [27] the rules proposed by Altschul [31]: a pair of homologs was retained only if they were separated by a distance less than 250 PAM units, and if each segment had a minimal size of 80 residues. Because this work focuses on orthologs belonging to synteny blocks, we further kept only the pairs of homologs where the alignment extended for at least 80% of the length of the shorter matching protein.

### Detecting orthologs by the RSD approach

Our Reciprocal Smallest Distance (RSD) approach takes advantage of specific properties of the DARWIN package [25,26]. Since AllAll program calculates the evolutionary distance separating homologous genes in PAM units and keeps the shortest distance for each analyzed pair of proteins, it was trivial to determine the best reciprocal-distance ortholog pairs in a comparison of two proteomes. Protein *a *encoded by genome G_A _and protein *b *encoded by genome G_B _are the best pair of orthologs only if the PAM distance separating *a *from *b *was smaller than that separating either *a *from any other protein encoded by G_B _and *b *from any other protein encoded by G_A_. We automated this search (Table 5, step 2a).

### Grouping homologs in consistent families

We used a two-step strategy for grouping the homologs into families. First, we grouped all homologs transitively, using a graph algorithm for detecting connected components. Step 2b (see Table 5) automatically gathers into one family all homologs that are related by a chain of similarities, collecting all relatives of both members of each pair until no further pairwise relationship is found.

Unfortunately, transitive grouping created many large, heterogeneous pseudo-families in which quite different closely-related protein groups were linked together by a single spurious edge, where one protein belonging to the first group appeared similar enough to one protein of the other group to form an apparent pair, which then transitively linked the two dissimilar groups into one family. Since this was clearly undesirable, we applied a second graph algorithm (implemented in a Perl graph library) for "bridge detection" (Table 5, step 2c). In graph theory, a bridge is defined as an edge whose removal disconnects the graph in two subclusters; removing such bridges allowed us to obtain much more homogeneous and correct families. In a third step (Table 5, step 2d), we broke additional unwanted links by applying the MCL (Markov Cluster) algorithm [33] to a very large aggregate of related homologs. MCL was used with an inflation value of 3.

### Reconstructing evolutionary trees for families of homologs

After assembling the families and filtering them for consistency as described above, we aligned their homologous sequences using the MUSCLE program [58]. Then, we processed these multiple alignments with a maximum likelihood approach to reconstruct the phylogeny of the corresponding family, using the PhyML software [59].

### Analyzing evolutionary trees to identify orthologs

Each tree is examined by *treeortho*, an algorithm (see Additional file 2) that examines each node (Table 5, step 2e), recursively spanning the tree starting from the mid-length root. As shown on a simple example (Fig. 1), each internal node, whichever its depth ("age"), is evaluated by analyzing its leaves, the present-day organisms. When the descendents of a node are in the same species, this node is interpreted as a gene duplication event (D, black circle). This is the case for the deepest node in the simplified tree adapted from a previous work on the evolutionary history of carbamoyltransferases [60], because the two branches stemming from this node display leaves that correspond to the same set of species. This defines two paralogous groups corresponding to present-day aspartate and ornithine carbamoyltransferases respectively. However, each time the descendents of a node are in separate species, it is a speciation event (S, gray diamond). Accordingly, it is possible to identify the sets of leaves that are orthologous to each other. If a duplication node is found in a proximal position, the two leaves will correspond to the so-called in-paralogs [32]. This is the case for genes *argF *and *argI *which appeared specifically in *E. coli *after the last speciation event. In this case, both in-paralogous proteins will be included in the set of orthologs.

### Creating a relational database for synteny data

The repository for all these data and results is a relational database we call SynteBase. It is implemented in PostgreSQL 8.1 [61], one of the most advanced open source databases, installed on a Linux platform. SynteBase is composed of five tables (see Additional file 4). Two tables contain primary data extracted from public databanks (GenBank/EMBL/DDBJ). Table *genome *holds information about the 107 studied genomes and table *protein *contains information about their 295,608 proteins, such as amino acid sequence, length, species name, location of encoding gene, etc. The three other tables, *ortho*, *neighbourpairs*, and *syntenic blocks*, contain the results of detection of orthologs and of pairs of adjacent orthologs, respectively. These three tables were populated after applying the computations described below and in the Results.

### Querying SynteBase to identify pairs of adjacent orthologs

Once we have determined the set of orthologs for each pair of genomes, we tried to identify how many belong to pairs of adjacent orthologs (Table 5, step 3a). To do that we filled out the *ortho *table and we applied the following SQL query based on a join operation of the *ortho *table with itself, selecting all rows where the orthologs of genes that are adjacent in genome 1 are on the same DNA strand and adjacent in genome 2:

Select * from orthologs as b1, orthologs as b2

Where b1.pidtemp_1 = (b2.pidtemp_1+1)

AND (b1.pidtemp_2 = (b2.pidtemp_2+1) OR b1.pidtemp_2 = (b2.pidtemp_2-1))

AND b1.strand_1 = b2.strand_1

AND b1.strand_2 = b2.strand_2;

In a second step, we attempted to extend the detected neighborhood relationships to larger synteny blocks (Table 5, step 3b). Blocks of size greater than 2 were detected by progressive accretion of blocks of size 2 that share a common pair of orthologs.

### Statistical analysis of the PAM distances of syntenic versus nonsyntenic genes in all genome comparisons

We call X1 the set of PAM distances separating orthologs located outside synteny blocks and X2 the set of PAM distances separating orthologs belonging to synteny blocks.

The hypothesis *H*_0_:*E*(*X*_1_) = *E*(*X*_2_) was tested against the hypothesis *H*_1 _: *E*(*X*_1_) > *E*(*X*_2_). Since we observed that the distribution of the PAM distances found in all intergenomic comparisons does not fit a Gaussian distribution, we preferred using a more robust bootstrap t-test [62] instead of a classical Student's t-test with n_1_+n_2_-2 freedom degrees, based on the statistics T=X¯1−X¯2−(E(X1)−E(X2))S^1n1+1n2
 MathType@MTEF@5@5@+=feaafiart1ev1aaatCvAUfKttLearuWrP9MDH5MBPbIqV92AaeXatLxBI9gBaebbnrfifHhDYfgasaacPC6xNi=xH8viVGI8Gi=hEeeu0xXdbba9frFj0xb9qqpG0dXdb9aspeI8k8fiI+fsY=rqGqVepae9pg0db9vqaiVgFr0xfr=xfr=xc9adbaqaaeGacaGaaiaabeqaaeqabiWaaaGcbaqaaiabdsfaujabd2da9KqbaoaalaaabaGafmiwaGLbaebadaWgaaqaaiabbgdaXaqabaGaeyOeI0IafmiwaGLbaebadaWgaaqaaiabbkdaYaqabaGaeyOeI0YaaeWaaeaacqWGfbqrdaqadaqaaiabdIfaynaaBaaabaGaeeymaedabeaaaiaawIcacaGLPaaacqGHsislcqWGfbqrdaqadaqaaiabdIfaynaaBaaabaGaeeOmaidabeaaaiaawIcacaGLPaaaaiaawIcacaGLPaaaaeaacuWGtbWugaqcamaakaaabaWaaSaaaeaacqqGXaqmaeaacqWGUbGBdaWgaaqaaiabbgdaXaqabaaaaiabdUcaRmaalaaabaGaeeymaedabaGaemOBa42aaSbaaeaacqqGYaGmaeqaaaaaaeqaaaaaaaaa@4A6E@, where S^
 MathType@MTEF@5@5@+=feaafiart1ev1aaatCvAUfKttLearuWrP9MDH5MBPbIqV92AaeXatLxBI9gBaebbnrfifHhDYfgasaacPC6xNi=xH8viVGI8Gi=hEeeu0xXdbba9frFj0xb9qqpG0dXdb9aspeI8k8fiI+fsY=rqGqVepae9pg0db9vqaiVgFr0xfr=xfr=xc9adbaqaaeGacaGaaiaabeqaaeqabiWaaaGcbaGafm4uamLbaKaaaaa@2D12@ is the standard deviation estimator of *T*. It is more accurate to estimate the quantiles of T from replicates of the studentized boostrap statisticsT∗(b)=X1∗(b)¯−X2∗(b)¯−(X1¯−X2¯)S∗(b)1 n1 +1 n2
 MathType@MTEF@5@5@+=feaafiart1ev1aaatCvAUfKttLearuWrP9MDH5MBPbIqV92AaeXatLxBI9gBaebbnrfifHhDYfgasaacPC6xNi=xH8viVGI8Gi=hEeeu0xXdbba9frFj0xb9qqpG0dXdb9aspeI8k8fiI+fsY=rqGqVepae9pg0db9vqaiVgFr0xfr=xfr=xc9adbaqaaeGacaGaaiaabeqaaeqabiWaaaGcbaqaaiabdsfaunaaCaaaleqabaGaey4fIOYaaeWaaeaacqWGIbGyaiaawIcacaGLPaaaaaGccqWG9aqpjuaGdaWcaaqaamaanaaabaGaemiwaG1aa0baaeaacqqGXaqmaeaacqGHxiIkdaqadaqaaiabdkgaIbGaayjkaiaawMcaaaaaaaGaeyOeI0Yaa0aaaeaacqWGybawdaqhaaqaaiabbkdaYaqaaiabgEHiQmaabmaabaGaemOyaigacaGLOaGaayzkaaaaaaaacqGHsisldaqadaqaamaanaaabaGaemiwaG1aaSbaaeaacqqGXaqmaeqaaaaacqGHsisldaqdaaqaaiabdIfaynaaBaaabaGaeeOmaidabeaaaaaacaGLOaGaayzkaaaabaGaem4uam1aaWbaaeqabaGaey4fIOYaaeWaaeaacqWGIbGyaiaawIcacaGLPaaaaaWaaOaaaeaadaWcaaqaaiabbgdaXiabbccaGaqaaiabd6gaUnaaBaaabaGaeeymaeJaeeiiaacabeaaaaGaem4kaSYaaSaaaeaacqqGXaqmcqqGGaaiaeaacqWGUbGBdaWgaaqaaiabbkdaYaqabaaaaaqabaaaaaaaaa@56FE@, where X1∗(b)
 MathType@MTEF@5@5@+=feaafiart1ev1aaatCvAUfKttLearuWrP9MDH5MBPbIqV92AaeXatLxBI9gBaebbnrfifHhDYfgasaacPC6xNi=xH8viVGI8Gi=hEeeu0xXdbba9frFj0xb9qqpG0dXdb9aspeI8k8fiI+fsY=rqGqVepae9pg0db9vqaiVgFr0xfr=xfr=xc9adbaqaaeGacaGaaiaabeqaaeqabiWaaaGcbaGaemiwaG1aa0baaSqaaiabbgdaXaqaaiabgEHiQmaabmaabaGaemOyaigacaGLOaGaayzkaaaaaaaa@31E7@ and X2∗(b)
 MathType@MTEF@5@5@+=feaafiart1ev1aaatCvAUfKttLearuWrP9MDH5MBPbIqV92AaeXatLxBI9gBaebbnrfifHhDYfgasaacPC6xNi=xH8viVGI8Gi=hEeeu0xXdbba9frFj0xb9qqpG0dXdb9aspeI8k8fiI+fsY=rqGqVepae9pg0db9vqaiVgFr0xfr=xfr=xc9adbaqaaeGacaGaaiaabeqaaeqabiWaaaGcbaGaemiwaG1aa0baaSqaaiabbkdaYaqaaiabgEHiQmaabmaabaGaemOyaigacaGLOaGaayzkaaaaaaaa@31E9@ are bootstrap samples, and S∗(b)=∑i=0n1(X1,i∗(b)−X1∗(b)¯)2+∑j=0n2(X2,j∗(b)−X2∗(b)¯)2n1+n2−2
 MathType@MTEF@5@5@+=feaafiart1ev1aaatCvAUfKttLearuWrP9MDH5MBPbIqV92AaeXatLxBI9gBaebbnrfifHhDYfgasaacPC6xNi=xH8viVGI8Gi=hEeeu0xXdbba9frFj0xb9qqpG0dXdb9aspeI8k8fiI+fsY=rqGqVepae9pg0db9vqaiVgFr0xfr=xfr=xc9adbaqaaeGacaGaaiaabeqaaeqabiWaaaGcbaqaaiabdofatnaaCaaaleqabaGaey4fIOYaaeWaaeaacqWGIbGyaiaawIcacaGLPaaaaaGccqWG9aqpjuaGdaGcaaqaamaalaaabaWaaabCaeaadaqadaqaaiabdIfaynaaDaaabaGaeeymaeJaeeilaWIaemyAaKgabaGaey4fIOYaaeWaaeaacqWGIbGyaiaawIcacaGLPaaaaaGaeyOeI0Yaa0aaaeaacqWGybawdaqhaaqaaiabbgdaXaqaaiabgEHiQmaabmaabaGaemOyaigacaGLOaGaayzkaaaaaaaaaiaawIcacaGLPaaadaahaaqabeaacqqGYaGmaaaabaGaemyAaKMaemypa0JaeeimaadabaGaemOBa4MaemymaedacqGHris5aiabgUcaRmaaqahabaWaaeWaaeaacqWGybawdaqhaaqaaiabbkdaYiabbYcaSiabdQgaQbqaaiabgEHiQmaabmaabaGaemOyaigacaGLOaGaayzkaaaaaiabgkHiTmaanaaabaGaemiwaG1aa0baaeaacqqGYaGmaeaacqGHxiIkdaqadaqaaiabdkgaIbGaayjkaiaawMcaaaaaaaaacaGLOaGaayzkaaWaaWbaaeqabaGaeeOmaidaaaqaaiabdQgaQjabd2da9iabbcdaWaqaaiabd6gaUjabdkdaYaGaeyyeIuoaaeaacqWGUbGBieaacqWFXaqmcqWGRaWkcqWGUbGBcqWFYaGmcqGHsislcqqGYaGmaaaabeaaaaaa@6E34@ is the standard deviation estimator of the bootstrap sample, and 1 ≤ *b *≤ 300.

Under H_0_, the law of T=X¯1−X¯2S^1n1+1n2
 MathType@MTEF@5@5@+=feaafiart1ev1aaatCvAUfKttLearuWrP9MDH5MBPbIqV92AaeXatLxBI9gBaebbnrfifHhDYfgasaacPC6xNi=xH8viVGI8Gi=hEeeu0xXdbba9frFj0xb9qqpG0dXdb9aspeI8k8fiI+fsY=rqGqVepae9pg0db9vqaiVgFr0xfr=xfr=xc9adbaqaaeGacaGaaiaabeqaaeqabiWaaaGcbaqaaiabdsfaujabd2da9KqbaoaalaaabaGafmiwaGLbaebadaWgaaqaaiabbgdaXaqabaGaeyOeI0IafmiwaGLbaebadaWgaaqaaiabbkdaYaqabaaabaGafm4uamLbaKaadaGcaaqaamaalaaabaGaeeymaedabaGaemOBa42aaSbaaeaacqqGXaqmaeqaaaaacqWGRaWkdaWcaaqaaiabbgdaXaqaaiabd6gaUnaaBaaabaGaeeOmaidabeaaaaaabeaaaaaaaaa@3D4B@ is well approximated by the sampling law of T* i.e. {T*^(*b*)^, *b *= 1 ... 300}.

To perform the test, we computed 300 bootstrap replicates of T* and calculated the empirical 95% quantile *q*_0.95_. H_0 _is rejected if T is greater than the quantile of T* at 95%, i.e. if *T *> *q*_0.95_. The test was applied to each intergenomic comparison.

## Authors' contributions

FL wrote the different programs necessary to collect and analyze data. The three authors participated in the design of the different experimental approaches, the conception of the different tools, and the data analysis. The three authors wrote together this manuscript.

## Supplementary Material

Additional File 1 **Table 1**. List of the 107 organisms (sorted by their taxonomy) used to compare gene order and to identify all orthologs. The complete taxonomy details and its abbreviated name are given for each studied organism. Click here for file

Additional File 2 Treeortho, an algorithm to identify orthologs in evolutionary trees. The whole annotated Perl code for distinguishing orthologs and paralogs. Click here for file

Additional File 3 A tree of CheC present in euryarchaeota and rooted with bacterial sequences. We used the full set of CheC sequences that are presently available in euryarchaeota. Click here for file

Additional File 5 **Table 2**. The longest synteny block in SynteBase. The whole set of adjacent POGs present in *Bacillus halodurans *and *Bacillus subtilis *forming this longest synteny block found in comparing the 107 organisms under study in this work. Click here for file

Additional File 4 **Figure 2**. Structure of SynteBase. The five tables of SynteBase and their respective links are detailed. Click here for file
