# Supplementary figures and images for: Assessing the evolutionary rate of positional orthologous genes in prokaryotes using synteny data
Source: BMC Evol Biol. 2007 Nov 29;7:237. doi: 10.1186/1471-2148-7-237 (PMC2238764; doi:10.1186/1471-2148-7-237)

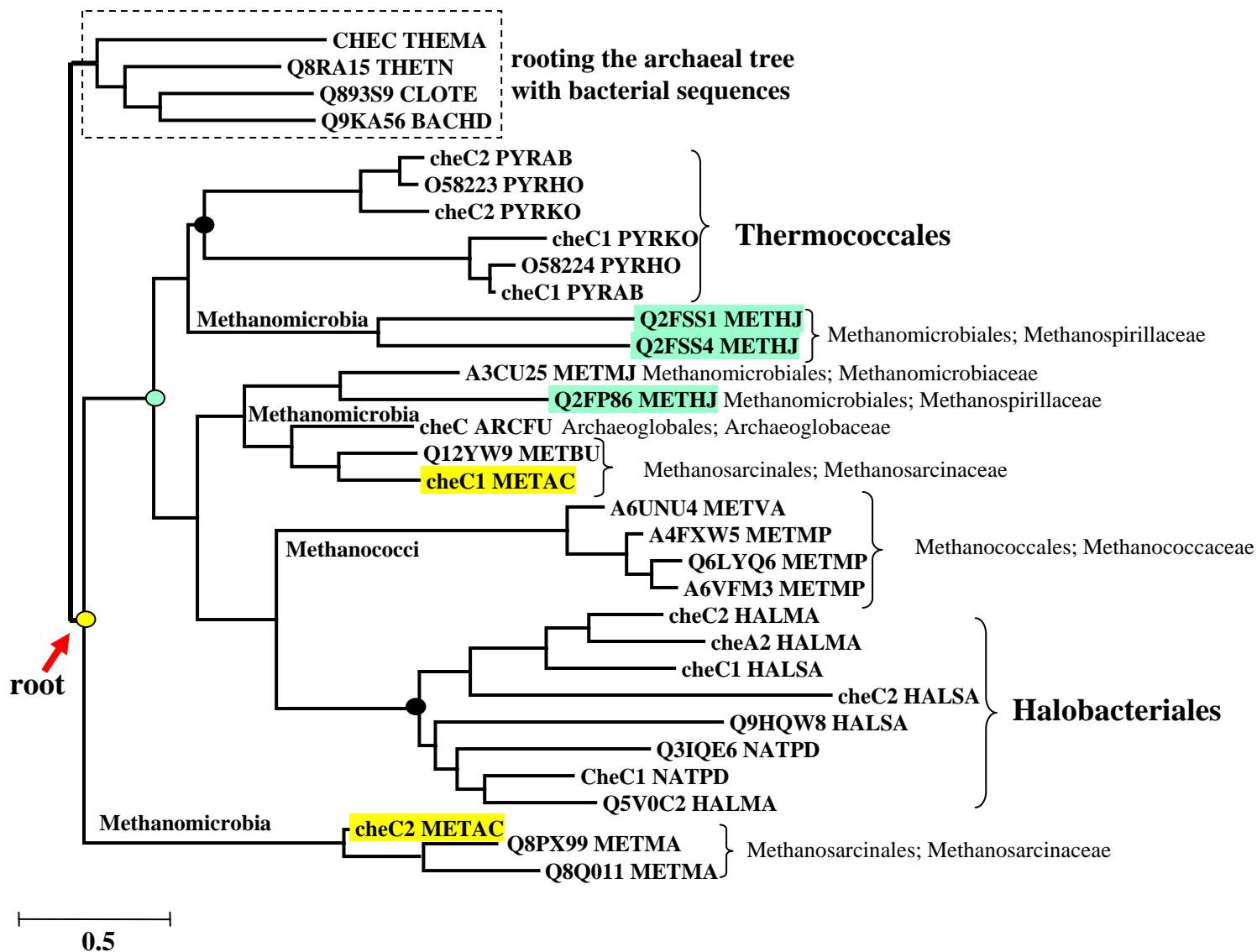

Supplement: Additional File 1 — Table 1. List of the 107 organisms (sorted by their taxonomy) used to compare gene order and to identify all orthologs. The complete taxonomy details and its abbreviated name are given for each studied organism. [file 1471-2148-7-237-S1.pdf]

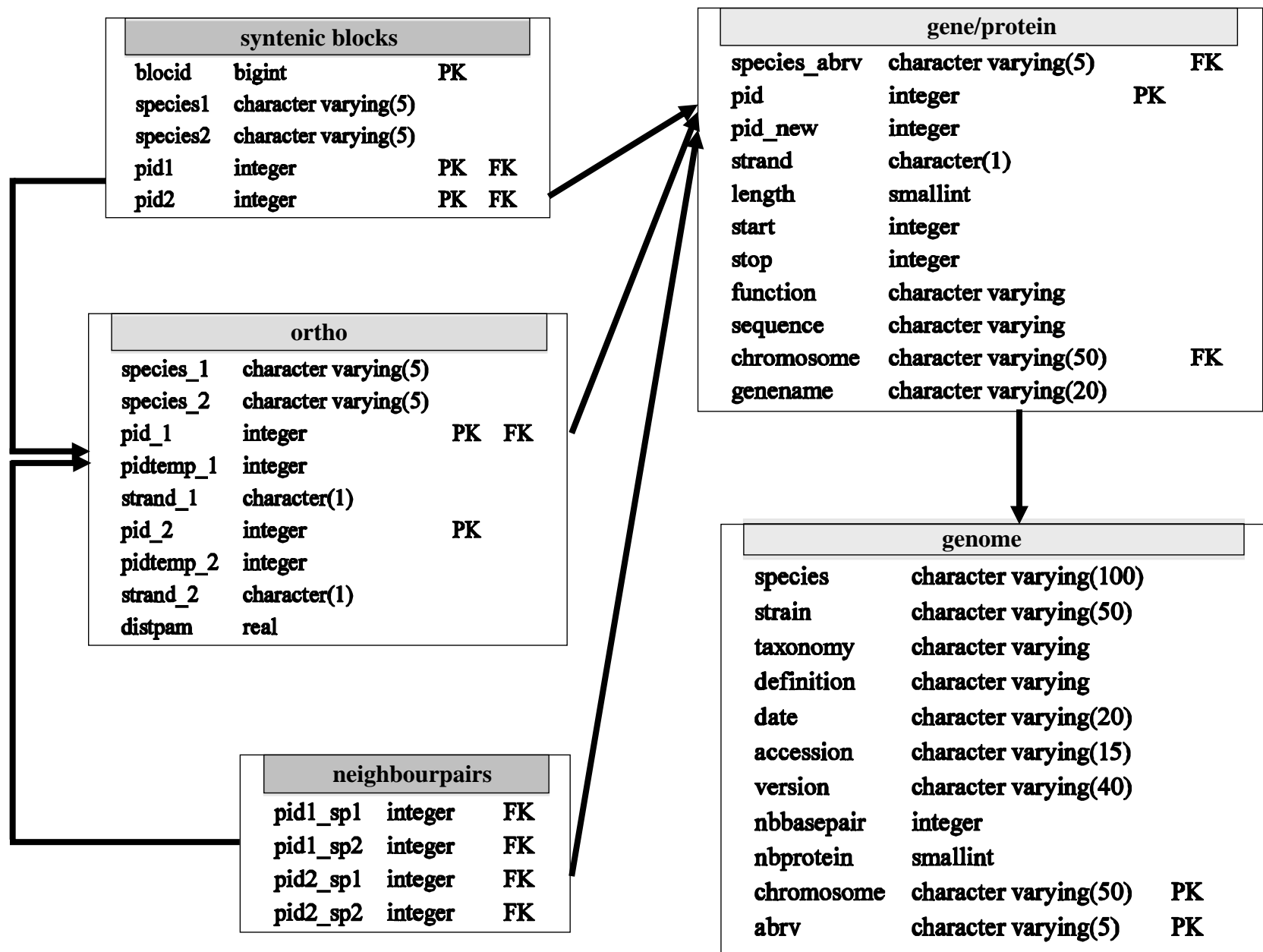

Sup\_Figure 1

Supplement: Additional File 2 — Treeortho, an algorithm to identify orthologs in evolutionary trees. The whole annotated Perl code for distinguishing orthologs and paralogs. [file 1471-2148-7-237-S2.pdf]
