# Supplementary material for: Assessing the evolutionary rate of positional orthologous genes in prokaryotes using synteny data
Source: BMC Evol Biol. 2007 Nov 29;7:237. doi: 10.1186/1471-2148-7-237 (PMC2238764; doi:10.1186/1471-2148-7-237)
Supplement: Additional File 3 — A tree of CheC present in euryarchaeota and rooted with bacterial sequences. We used the full set of CheC sequences that are presently available in euryarchaeota. [file 1471-2148-7-237-S3.pdf]

Treeortho, an algorithm to identify orthologs in evolutionary trees

```
function parcourOrtho(Node node) :
if(duplication(node)){
	foreach child (enfantsD(node){
	parcourOrtho(child);
	}
}
else{
	children = children(node);
	// We traverse all possible pairs of children of 'node'
	for(i = 0 ; i < length(children) ; i++){
	  for(j = i+1 ; j <= length(children) ; j++){
		// All the pairs of proteins are orthologous
		// Because there is no duplication
		foreach child1 ( leaves(children[$i]) ){
			foreach child2(leaves(children[$j]) ){
				print "ortho child1-child2]";
			}
		}
	  }
	}
	foreach child (children){
	  parcourOrtho(child);
	}
}

function duplication(Node node) :
// If the node is leaf, it's not a duplication
if(leaf(node)){
	return FALSE;
}

children = children(node);
intersect = FALSE;
// We traverse the direct children of node by pairs
for(i=0;i<length(children) && !$intersect;$i++){
	for(j=i+1;j<=length(children) && !$intersect;$j++){
		if(intersect(leaves(children[$i]),leaves(children[$j])){
			$intersect = TRUE ;
```
