# Supplementary material for: Assessing the evolutionary rate of positional orthologous genes in prokaryotes using synteny data
Source: BMC Evol Biol. 2007 Nov 29;7:237. doi: 10.1186/1471-2148-7-237 (PMC2238764; doi:10.1186/1471-2148-7-237)
Supplement: Additional File 5 — Table 2. The longest synteny block in SynteBase. The whole set of adjacent POGs present in Bacillus halodurans and Bacillus subtilis forming this longest synteny block found in comparing the 107 organisms under study in this work. [file 1471-2148-7-237-S5.pdf]

species	pid	gene	function	species	pid	gene	function
**Bacha**	**15614990**	***rpsB***	**30S ribosomal protein S2**	**Bacsu**	**16078712**	***rpsB***	**ribosomal protein S2**
**Bacha**	**15614989**	***tsf***	**elongation factor Ts**	**Bacsu**	**16078713**	***tsf***	**elongation factor Ts**
**Bacha**	**15614988**	***smbA***	**uridylate kinase**	**Bacsu**	**16078714**	***smbA***	**uridylate kinase**
**Bacha**	**15614987**	***frr***	**ribosome recycling factor**	**Bacsu**	**16078715**	***frr***	**ribosome recycling factor**
**Bacha**	**15614986**	***uppS***	**undecaprenyl pyrophosphate synthetase**	**Bacsu**	**16078716**	***yluA***	**_**
**Bacha**	**15614985**	***cdsA***	**phosphatidate cytidylyltransferase**	**Bacsu**	**16078717**	***cdsA***	**phosphatidate cytidylyltransferase (CDP-diglyceride synthase)**
**Bacha**	**15614984**	***BH2421***	**1-deoxy-d-xylulose-5-phosphate reductoisomerase**	**Bacsu**	**16078718**	***yluB***	**_**
**Bacha**	**15614983**	***BH2420***	**_**	**Bacsu**	**16078719**	***yluC***	**_**
**Bacha**	**15614982**	***proS***	**prolyl-tRNA synthetase**	**Bacsu**	**16078720**	***proS***	**prolyl-tRNA synthetase**
**Bacha**	**15614981**	***polC***	**DNA polymerase III (alpha subunit)**	**Bacsu**	**16078721**	***polC***	**DNA polymerase III (alpha subunit)**
**Bacha**	**15614980**	***BH2417***	**_**	**Bacsu**	**16078722**	***ylxS***	**_**
**Bacha**	**15614979**	***nusA***	**transcriptional terminator**	**Bacsu**	**16078723**	***nusA***	**_**
**Bacha**	**15614978**	***BH2415***	**_**	**Bacsu**	**16078724**	***ylxR***	**_**
**Bacha**	**15614977**	***BH2414***	**_**	**Bacsu**	**16078725**	***ylxQ***	**_**
**Bacha**	**15614976**	***infB***	**translation initiation factor IF-2**	**Bacsu**	**16078726**	***infB***	**initiation factor IF-2**
**Bacha**	**15614975**	***BH2412***	**_**	**Bacsu**	**16078727**	***ylxP***	**_**
**Bacha**	**15614974**	***rbfA***	**ribosome-binding factor A**	**Bacsu**	**16078728**	***rbfA***	**ribosome-binding factor A**
**Bacha**	**15614973**	***truB***	**tRNA pseudouridine 5S synthase**	**Bacsu**	**16078729**	***truB***	**tRNA pseudouridine 55 synthase**
**Bacha**	**15614972**	***ribC***	**riboflavin kinase / FAD synthase**	**Bacsu**	**16078730**	***ribC***	**riboflavin kinase**
**Bacha**	**15614971**	***rpsO***	**30S ribosomal protein S15**	**Bacsu**	**16078731**	***rpsO***	**ribosomal protein S15 (BS18)**
**Bacha**	**15614970**	***pnpA***	**polynucleotide phosphorylase**	**Bacsu**	**16078732**	***pnpA***	**polynucleotide phosphorylase (PNPase)**
**Bacha**	**15614969**	***BH2406***	**_**	**Bacsu**	**16078733**	***ylxY***	**_**
**Bacha**	**15614968**	***BH2405***	**processing protease**	**Bacsu**	**16078734**	***ymxG***	**_**
**Bacha**	**15614967**	***BH2404***	**_**	**Bacsu**	**16078735**	***ymxH***	**_**
**Bacha**	**15614966**	***spoVFA***	**dipicolinate synthase subunit A**	**Bacsu**	**16078736**	***spoVFA***	**dipicolinate synthase subunit A**
**Bacha**	**15614965**	***spoVFB***	**dipicolinate synthase subunit B**	**Bacsu**	**16078737**	***spoVFB***	**dipicolinate synthase subunit B**
**Bacha**	**15614964**	***asd***	**aspartate-semialdehyde dehydrogenase**	**Bacsu**	**16078738**	***asd***	**aspartate-semialdehyde dehydrogenase**
**Bacha**	**15614963**	***dapG***	**aspartokinase I (alpha and beta subunits)**	**Bacsu**	**16078739**	***dapG***	**aspartokinase I (alpha and beta subunits)**
**Bacha**	**15614962**	***dapA***	**dihydrodipicolinate synthase**	**Bacsu**	**16078740**	***dapA***	**dihydrodipicolinate synthase**
**Bacha**	**15614961**	***BH2398***	**_**	**Bacsu**	**16078741**	***ymfA***	**_**

Table 2. The longest synteny block in SynteBase.
Bacha = *Bacillus halodurans*
Bacsu = *Bacillus subtilis*
