# Supplementary material for: Assessing the evolutionary rate of positional orthologous genes in prokaryotes using synteny data
Source: BMC Evol Biol. 2007 Nov 29;7:237. doi: 10.1186/1471-2148-7-237 (PMC2238764; doi:10.1186/1471-2148-7-237)
Supplement: Additional File 4 — Figure 2. Structure of SynteBase. The five tables of SynteBase and their respective links are detailed. [file 1471-2148-7-237-S4.pdf]

Abbv	Domain	phylum	class	Order	family	species name
Aerpe	Archaea	Crenarchaeota	Thermoprotei	Desulfurococcales	Desulfurococcaceae	*Aeropyrum pernix*
Sulso	Archaea	Crenarchaeota	Thermoprotei	Sulfolobales	Sulfolobaceae	*Sulfolobus solfataricus*
Pyrae	Archaea	Crenarchaeota	Thermoprotei	Thermoproteales	Thermoproteaceae	*Pyrobaculum aerophilum*
Arcfu	Archaea	Euryarchaeota	Archaeoglobi	Archaeoglobales	Archaeoglobaceae	*Archaeoglobus fulgidus*
Halma	Archaea	Euryarchaeota	Halobacteria	Halobacteriales	Halobacteriaceae	*Haloarcula marismortui*
Halob	Archaea	Euryarchaeota	Halobacteria	Halobacteriales	Halobacteriaceae	*Halobacterium sp*
Metth	Archaea	Euryarchaeota	Methanobacteria	Methanobacteriales	Methanobacteriaceae	*Methanothermobacter thermautotrophicus*
Metja	Archaea	Euryarchaeota	Methanococci	Methanococcales	Methanocaldococcaceae	*Methanocaldococcus jannaschii*
Metac	Archaea	Euryarchaeota	Methanomicrobia	Methanosarcinales	Methanosarcinaceae	*Methanosarcina acetivorans*
Metka	Archaea	Euryarchaeota	Methanopyri	Methanopyrales	Methanopyraceae	*Methanopyrus kandleri*
Theko	Archaea	Euryarchaeota	Thermococci	Thermococcales	Thermococcaceae	*Thermococcus kodakarensis*
Pyrab	Archaea	Euryarchaeota	Thermococci	Thermococcales	Thermococcaceae	*Pyrococcus abyssi*
Picto	Archaea	Euryarchaeota	Thermoplasmata	Thermoplasmatales	Picrophilaceae	*Picrophilus torridus*
Theac	Archaea	Euryarchaeota	Thermoplasmata	Thermoplasmatales	Thermoplasmataceae	*Thermoplasma acidophilum*
Naneq	Archaea	Nanoarchaeota	-	-	-	*Nanoarchaeum equitans*
Symth	Bacteria	Actinobacteria	Actinobacteria	-	-	*Symbiobacterium thermophilum*
Trowh	Bacteria	Actinobacteria	Actinobacteria	Actinomycetales	Cellulomonadaceae	*Tropheryma whipplei*
Corgl	Bacteria	Actinobacteria	Actinobacteria	Actinomycetales	Corynebacteriaceae	*Corynebacterium glutamicum*
Leixy	Bacteria	Actinobacteria	Actinobacteria	Actinomycetales	Microbacteriaceae	*Leifsonia xyli*
Mycle	Bacteria	Actinobacteria	Actinobacteria	Actinomycetales	Mycobacteriaceae	*Mycobacterium leprae*
Myctu	Bacteria	Actinobacteria	Actinobacteria	Actinomycetales	Mycobacteriaceae	*Mycobacterium tuberculosis*
Strco	Bacteria	Actinobacteria	Actinobacteria	Actinomycetales	Streptomycetaceae	*Streptomyces coelicolor*
Biflo	Bacteria	Actinobacteria	Actinobacteria	Bifidobacteriales	Bifidobacteriaceae	*Bifidobacterium longum*
Aquae	Bacteria	Aquificae	Aquificae	Aquificales	Aquificaceae	*Aquifex aeolicus*
Bacth	Bacteria	B/C[1]	Bacteroides	Bacteroidales	Bacteroidaceae	*Bacteroides thetaiotaomicron*
Porgi	Bacteria	B/C[1]	Bacteroides	Bacteroidales	Porphyromonadaceae	*Porphyromonas gingivalis*
Chlte	Bacteria	B/C[1]	Chlorobia	Chlorobiales	Chlorobiaceae	*Chlorobium tepidum*
Chlmu	Bacteria	C/V[2]	Chlamydiae	Chlamydiales	Chlamydiaceae	*Chlamydia muridarum*
Chltr	Bacteria	C/V[2]	Chlamydiae	Chlamydiales	Chlamydiaceae	*Chlamydia trachomatis*
Chlpn	Bacteria	C/V[2]	Chlamydiae	Chlamydiales	Chlamydiaceae	*Chlamydophila pneumoniae*
Parac	Bacteria	C/V[2]	Chlamydiae	Chlamydiales	Parachlamydiaceae	*Parachlamydia species*
Dehet	Bacteria	Chloroflexi	Dehalococcoidetes	-	-	*Dehalococcoides ethenogenes*
Glovi	Bacteria	cyanobacteria	-	Chroococcales	-	*Gloeobacter violaceus*
Synec	Bacteria	cyanobacteria	-	Chroococcales	-	*Synechocystis species*

**Theel**	**Bacteria**	**cyanobacteria**	**-**	**Chroococcales**	**-**	***Thermosynechococcus elongatus***
**Nosto**	**Bacteria**	**cyanobacteria**	**-**	**Nostocales**	**Nostocaceae**	***Nostoc species***
**Proma**	**Bacteria**	**cyanobacteria**	**-**	**Prochlorophytes**	**Prochlorococcaceae**	***Prochlorococcus marinus***
**PromM**	**Bacteria**	**cyanobacteria**	**-**	**Prochlorophytes**	**Prochlorococcaceae**	***Prochlorococcus marinus***
**Deira**	**Bacteria**	**Deinococcus-Thermus**	**Deinococci**	**Deinococcales**	**Deinococcaceae**	***Deinococcus radiodurans***
**Theth**	**Bacteria**	**Deinococcus-Thermus**	**Deinococci**	**Thermales**	**Thermaceae**	***Thermus thermophilus***
**Bacha**	**Bacteria**	**Firmicutes**	**Bacilli**	**Bacillales**	**Bacillaceae**	***Bacillus halodurans***
**Bacsu**	**Bacteria**	**Firmicutes**	**Bacilli**	**Bacillales**	**Bacillaceae**	***Bacillus subtilis***
**Oceih**	**Bacteria**	**Firmicutes**	**Bacilli**	**Bacillales**	**Bacillaceae**	***Oceanobacillus iheyensis***
**Lisin**	**Bacteria**	**Firmicutes**	**Bacilli**	**Bacillales**	**Listeriaceae**	***Listeria innocua***
**Staau**	**Bacteria**	**Firmicutes**	**Bacilli**	**Bacillales**	**Staphylococcaceae**	***Staphylococcus aureus***
**Entfa**	**Bacteria**	**Firmicutes**	**Bacilli**	**Lactobacillales**	**Enterococcaceae**	***Enterococcus faecalis***
**Lacla**	**Bacteria**	**Firmicutes**	**Bacilli**	**Lactobacillales**	**Streptococcaceae**	***Lactococcus lactis***
**Strpn**	**Bacteria**	**Firmicutes**	**Bacilli**	**Lactobacillales**	**Streptococcaceae**	***Streptococcus pneumoniae***
**Strpy**	**Bacteria**	**Firmicutes**	**Bacilli**	**Lactobacillales**	**Streptococcaceae**	***Streptococcus pyogenes***
**Cloac**	**Bacteria**	**Firmicutes**	**Clostridia**	**Clostridiales**	**Clostridiaceae**	***Clostridium acetobutylicum***
**Clote**	**Bacteria**	**Firmicutes**	**Clostridia**	**Clostridiales**	**Clostridiaceae**	***Clostridium tetani***
**Thete**	**Bacteria**	**Firmicutes**	**Clostridia**	**Thermoanaerobacteriales**	**Thermoanaerobacteriaceae**	***Thermoanaerobacter tengcongensis***
**Oniye**	**Bacteria**	**Firmicutes**	**Mollicutes**	**Acholeplasmatales**	**Acholeplasmataceae**	***Phytoplasma asteris***
**Mycge**	**Bacteria**	**Firmicutes**	**Mollicutes**	**Mycoplasmatales**	**Mycoplasmataceae**	***Mycoplasma genitalium***
**Mycpe**	**Bacteria**	**Firmicutes**	**Mollicutes**	**Mycoplasmatales**	**Mycoplasmataceae**	***Mycoplasma penetrans***
**Mycpn**	**Bacteria**	**Firmicutes**	**Mollicutes**	**Mycoplasmatales**	**Mycoplasmataceae**	***Mycoplasma pneumoniae***
**Mycpu**	**Bacteria**	**Firmicutes**	**Mollicutes**	**Mycoplasmatales**	**Mycoplasmataceae**	***Mycoplasma pulmonis***
**Ureur**	**Bacteria**	**Firmicutes**	**Mollicutes**	**Mycoplasmatales**	**Mycoplasmataceae**	***Ureaplasma parvum***
**Mesfl**	**Bacteria**	**Firmicutes**	**Mollicutes**	**Entomoplasmatales**	**Entomoplasmataceae**	***Mesoplasma florum***
**Fusnu**	**Bacteria**	**Fusobacteria**	**Fusobacteria**	**Fusobacterales**	**Fusobacteriaceae**	***Fusobacterium nucleatum***
**Pirel**	**Bacteria**	**Planctomycetes**	**Planctomycetacia**	**Planctomycetales**	**Planctomycetaceae**	***Rhodopirellula baltica***
**Caucr**	**Bacteria**	**proteobacteria**	**alpha**	**Caulobacterales**	**Caulobacteraceae**	***Caulobacter crescentus***
**Braja**	**Bacteria**	**proteobacteria**	**alpha**	**Rhizobiales**	**Bradyrhizobiaceae**	***Bradyrhizobium japonicum***
**Rhopa**	**Bacteria**	**proteobacteria**	**alpha**	**Rhizobiales**	**Bradyrhizobiaceae**	***Rhodopseudomonas palustris***
**Brume**	**Bacteria**	**proteobacteria**	**alpha**	**Rhizobiales**	**Brucellaceae**	***Brucella melitensis***
**Meslo**	**Bacteria**	**proteobacteria**	**alpha**	**Rhizobiales**	**Phyllobacteriaceae**	***Mesorhizobium loti***
**Agrtu**	**Bacteria**	**proteobacteria**	**alpha**	**Rhizobiales**	**Rhizobiaceae**	***Agrobacterium tumefaciens***
**Sinme**	**Bacteria**	**proteobacteria**	**alpha**	**Rhizobiales**	**Rhizobiaceae**	***Sinorhizobium meliloti***
**Anama**	**Bacteria**	**proteobacteria**	**alpha**	**Rickettsiales**	**Anaplasmataceae**	***Anaplasma marginale***
**Ricco**	**Bacteria**	**proteobacteria**	**alpha**	**Rickettsiales**	**Rickettsiaceae**	***Rickettsia conorii***

**Ricpr**	**Bacteria**	**proteobacteria**	**alpha**	**Rickettsiales**	**Rickettsiaceae**	***Rickettsia prowazekii***
**Wolba**	**Bacteria**	**proteobacteria**	**alpha**	**Rickettsiales**	**Rickettsiaceae**	***Wolbachia pipientis***
**Zymmo**	**Bacteria**	**proteobacteria**	**alpha**	**Sphingomonadales**	**Sphingomonadaceae**	***Zymomonas mobilis***
**Borbr**	**Bacteria**	**proteobacteria**	**beta**	**Burkholderiales**	**Alcaligenaceae**	***Bordetella bronchiseptica***
**Ralso**	**Bacteria**	**proteobacteria**	**beta**	**Burkholderiales**	**Burkholderiaceae**	***Ralstonia solanacearum***
**Neime**	**Bacteria**	**proteobacteria**	**beta**	**Neisseriales**	**Neisseriaceae**	***Neisseria meningitidis***
**Niteu**	**Bacteria**	**proteobacteria**	**beta**	**Nitrosomonadales**	**Nitrosomonadaceae**	***Nitrosomonas europaea***
**Bdeba**	**Bacteria**	**proteobacteria**	**delta**	**Bdellovibrionales**	**Bdellovibrionaceae**	***Bdellovibrio bacteriovorus***
**Desps**	**Bacteria**	**proteobacteria**	**delta**	**Desulfobacterales**	**Desulfobulbaceae**	***Desulfotalea psychrophila***
**Desvu**	**Bacteria**	**proteobacteria**	**delta**	**Desulfovibrionales**	**Desulfovibrionaceae**	***Desulfovibrio vulgaris***
**Geosu**	**Bacteria**	**proteobacteria**	**delta**	**Desulfuromonadales**	**Geobacteraceae**	***Geobacter sulfurreducens***
**Sheon**	**Bacteria**	**proteobacteria**	**gamma**	**Alteromonadales**	**Alteromonadaceae**	***Shewanella oneidensis***
**Blofl**	**Bacteria**	**proteobacteria**	**gamma**	**Enterobacteriales**	**Enterobacteriaceae**	***Blochmannia floridanus***
**Buchn**	**Bacteria**	**proteobacteria**	**gamma**	**Enterobacteriales**	**Enterobacteriaceae**	***Buchnera aphidicola***
**Escco**	**Bacteria**	**proteobacteria**	**gamma**	**Enterobacteriales**	**Enterobacteriaceae**	***Escherichia coli***
**Salen**	**Bacteria**	**proteobacteria**	**gamma**	**Enterobacteriales**	**Enterobacteriaceae**	***Salmonella enterica***
**Wiggl**	**Bacteria**	**proteobacteria**	**gamma**	**Enterobacteriales**	**Enterobacteriaceae**	***Wigglesworthia glossinidia***
**Yerpe**	**Bacteria**	**proteobacteria**	**gamma**	**Enterobacteriales**	**Enterobacteriaceae**	***Yersinia pestis***
**Coxbu**	**Bacteria**	**proteobacteria**	**gamma**	**Legionellales**	**Coxiellaceae**	***Coxiella burnetii***
**Haein**	**Bacteria**	**proteobacteria**	**gamma**	**Pasteurellales**	**Pasteurellaceae**	***Haemophilus influenzae***
**Pasmu**	**Bacteria**	**proteobacteria**	**gamma**	**Pasteurellales**	**Pasteurellaceae**	***Pasteurella multocida***
**Acine**	**Bacteria**	**proteobacteria**	**gamma**	**Pseudomonadales**	**Moraxellaceae**	***Acinetobacter species***
**Pseae**	**Bacteria**	**proteobacteria**	**gamma**	**Pseudomonadales**	**Pseudomonadaceae**	***Pseudomonas aeruginosa***
**Fratu**	**Bacteria**	**proteobacteria**	**gamma**	**Thiotrichales**	**Francisellaceae**	***Francisella tularensis***
**Vibch**	**Bacteria**	**proteobacteria**	**gamma**	**Vibrionales**	**Vibrionaceae**	***Vibrio cholerae***
**Vibpa**	**Bacteria**	**proteobacteria**	**gamma**	**Vibrionales**	**Vibrionaceae**	***Vibrio parahaemolyticus***
**Xanax**	**Bacteria**	**proteobacteria**	**gamma**	**Xanthomonadales**	**Xanthomonadaceae**	***Xanthomonas axonopodis***
**Xylfa**	**Bacteria**	**proteobacteria**	**gamma**	**Xanthomonadales**	**Xanthomonadaceae**	***Xylella fastidiosa***
**Camje**	**Bacteria**	**proteobacteria**	**epsilon**	**Campylobacterales**	**Campylobacteraceae**	***Campylobacter jejuni***
**HelpJ**	**Bacteria**	**proteobacteria**	**epsilon**	**Campylobacterales**	**Helicobacteraceae**	***Helicobacter pylori***
**Helpy**	**Bacteria**	**proteobacteria**	**epsilon**	**Campylobacterales**	**Helicobacteraceae**	***Helicobacter pylori***
**Wolsu**	**Bacteria**	**proteobacteria**	**epsilon**	**Campylobacterales**	**Helicobacteraceae**	***Wolinella succinogenes***
**Lepin**	**Bacteria**	**Spirochaetes**	**Spirochaetes**	**Spirochaetales**	**Leptospiraceae**	***Leptospira interrogans***
**Borbu**	**Bacteria**	**Spirochaetes**	**Spirochaetes**	**Spirochaetales**	**Spirochaetaceae**	***Borrelia burgdorferi***
**Trede**	**Bacteria**	**Spirochaetes**	**Spirochaetes**	**Spirochaetales**	**Spirochaetaceae**	***Treponema denticola***
**Trepa**	**Bacteria**	**Spirochaetes**	**Spirochaetes**	**Spirochaetales**	**Spirochaetaceae**	***Treponema pallidum***
**Thema**	**Bacteria**	**Thermotogae**	**Thermotogae**	**Thermotogales**	**Thermotogaceae**	***Thermotoga maritima***

Table 1. List of the organisms (sorted by their taxonomy) used to compare gene order and to identify all orthologs.

[1]B/C = Bacteroidetes/Chlorobi group

[2]C/V = Chlamydiae/Verrucomicrobia group
